# Supplementary material for: DNase-1 Treatment Exerts Protective Effects in Neurogenic Pulmonary Edema via Regulating the Neutrophil Extracellular Traps after Subarachnoid Hemorrhage in Mice
Source: J Clin Med. 2022 Jul 27;11(15):4349. doi: 10.3390/jcm11154349 (PMC9369252; doi:10.3390/jcm11154349)
Supplement: Supplementary file 1 [file jcm-11-04349-s001.zip › Table1 S1.pdf]

	**Forward**（5’-3’）	**Reverse**（5’-3’）
TNF-α	ATCCGCGACGTGGAACTG	ACCGCCTGGAGTTCTGGAA
IL-1β	TTGTTCATCTCGGAGCCTGTA	AGCACCTTCTTTTCCTTCATC
IL-6	GCACTAGGTTTGCCGAGTAGA	AAGCTGGAGTCACAGAAGGAG
IL-10	TGCACTACCAAAGCCACAA	TAAGAGCAGGCAGCATAGCAG
β-Actin	GAGACCTTCAACACCCCAGC	CCACAGGATTCCATACCCAA
