# Supplementary figures and images for: DNase-1 Treatment Exerts Protective Effects in Neurogenic Pulmonary Edema via Regulating the Neutrophil Extracellular Traps after Subarachnoid Hemorrhage in Mice
Source: J Clin Med. 2022 Jul 27;11(15):4349. doi: 10.3390/jcm11154349 (PMC9369252; doi:10.3390/jcm11154349)

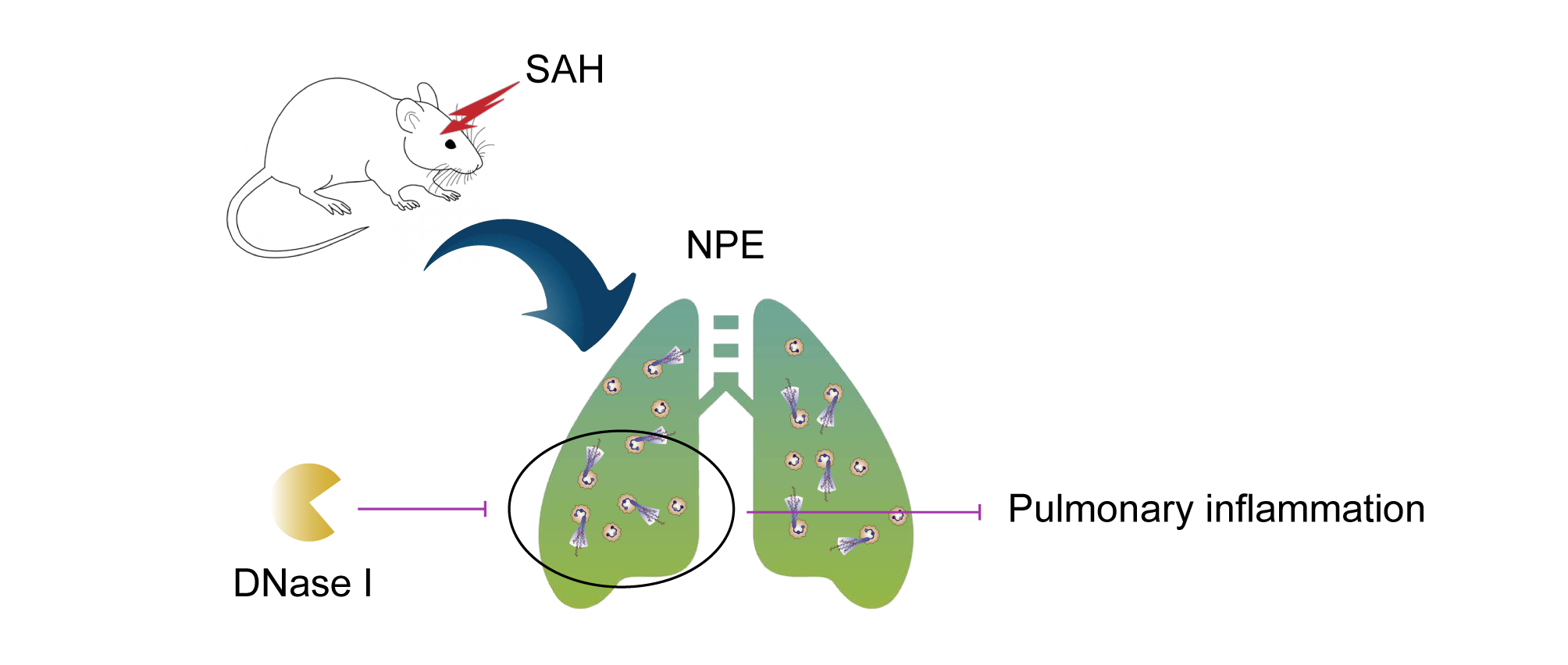

Supplement: Supplementary file 1 [file jcm-11-04349-s001.zip › Figure S1.tif]

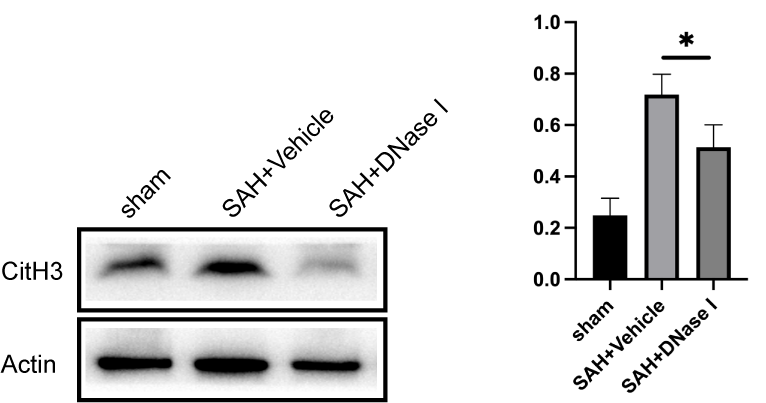

Supplement: Supplementary file 1 [file jcm-11-04349-s001.zip › Figure S2.tif]
